# Supplementary material for: In vivo imaging and pharmacokinetics of percutaneously injected ultrasound and X-ray imageable thermosensitive hydrogel loaded with doxorubicin versus free drug in swine
Source: PLoS One. 2024 Dec 19;19(12):e0310345. doi: 10.1371/journal.pone.0310345 (PMC11658602; doi:10.1371/journal.pone.0310345)
Supplement: S1 Appendix — (DOCX) [file pone.0310345.s001.docx]

**S1. Appendix. Materials and methods, image processing codes and supplemental figures.**

**Materials and methods**

**Chemicals.**

1,2-distearoyl-sn-glycero-3-phosphocholine (18:0 DSPC) (850365C-1g; Avanti lipids, Alabaster, Alabama, USA) and 0.4mL of 10mg/mL of Polyethylene glycol (PEG) 40 stearate (P3440-250G), both in chloroform (372978-1L) (Sigma Aldrich, Inc; Saint Louis, MO, USA). Normal saline (114-055-101, Quality Biological, Gaithersburg, Maryland, USA), glycerol (G7757-500ml), and propylene glycol (81380-1L) (Sigma Aldrich). Perfluorobutane gas (001836-100G) was obtained from Matrix Scientific, Columbia, SC, USA. Poloxamer 407, purified non-ionic (16758-250G) was obtained from Sigma Aldrich. Iodixanol (1123771), Visipaque 320 mg/mL, was obtained from GE Healthcare, Waukesha, WI, USA. Doxorubicin, Hydrochloride Salt, >99% (D-4000) was obtained from LC Laboratories, Woburn, Massachusetts, USA. Intramuscular ketamine (NC-0256) (25 mg/Kg), and midazolam (NC-0534) (0.5 mg/Kg) were obtained from Next Gen, Weatherford, Texas. Glycopyrrolate (0.01 mg/Kg) (NDC 0517-4620-25, American Regent, INC, Shirley, New York, USA) and anesthetized with propofol (1 mg/Kg intravenous) (A18293, Adooq Bioscience LLC, Irvine, California, USA) and maintained under general anesthesia with isoflurane (1-5%, Isoflo, Abbott Animal Health, North Chicago, IL). Beuthanasia-D (pentobarbital sodium 390 mg/mL and phenytoin sodium 50 mg/mL) (NDC 0061-0473-05) was obtained from Merck, New Jersey, USA. 2-mercaptoethanol was obtained from Sigma Aldrich. 0.1% trifluoroacetic acid (AC325330100) was obtained from Thermo Fisher Scientific, Waltham, Massachusetts), and acetonitrile (100665) was obtained from Sigma Aldrich. Daunorubicin (1164700-200MG) and Doxorubicin (1225703-50 MG) for calibration standards were obtained from Sigma Aldrich. Potassium phosphate monobasic (KH₂PO₄) and Zinc Sulfate monohydrate (ZnSO_4_*H_2_O) were obtained also from Sigma Aldrich.

**Matlab codes:**

**Calculation of area, circularity, and solidity**

close all

clear all

I=imread('Slide4.jpg');

I=rgb2gray(I);

imshow(I)

th = graythresh(I);

IcropEqTh = im2bw(I,th);

for i = 1

Icrop = imcrop(IcropEqTh) ;

imshow(Icrop)

stats(:,i) = regionprops(Icrop,'Area','Perimeter','Solidity','MajorAxisLength','MinorAxisLength','Orientation','Circularity');

end

**Calculation of entropy**

% Load the image (Replace 'your_image.jpg' with your actual image file)

img = imread('Slide23.jpg');

% Convert to grayscale if the image is RGB

if size(img, 3) == 3

img_gray = rgb2gray(img);

else

img_gray = img;

end

% Display the image and select ROI manually using a polygon

figure;

imshow(img_gray);

title('Draw a polygon around the ROI and double click when done');

roi = drawpolygon('Label', 'Select ROI', 'Color', 'r');

% Wait for the ROI to be finalized

wait(roi);

% Create a binary mask for the ROI

mask = createMask(roi);

% Extract the ROI using the mask

selectedROI = img_gray;

selectedROI(~mask) = 0; % Set pixels outside the ROI to 0

% Calculate the entropy of the selected ROI

% Entropy calculation only for the masked region

maskedValues = selectedROI(mask); % Extract non-zero (ROI) pixel values

roiEntropy = entropy(maskedValues);

% Display the entropy

fprintf('Entropy of the selected ROI: %f\n', roiEntropy);

**Color-coded contour plots**

% close all;

clear all;

% %CHANGE THESE INTENSITY and CONCENTRATION VALUES AS NEEDED!

% Image intensity values

levelList = [0 50 100 150 200];

% Injection time mL (should be same length as levelList)

injectionList= [0 1 2 3 4];

% Read in you image

I = imread('combozoom.jpg');

I2 = imread('combozoom.jpg');

% Image processing starts here!

%convert to grayscale

Igray = rgb2gray(I);

Igray2 = rgb2gray(I2);

figure('Position', [10 50 900 900])

ax(1) = subplot(3,2,1);

imshow(I);

title('1. Original Image')

% Histogram of gray scale and cropped plot

% YOU CAN USE THIS TO DETERMINE LEVELS

ax(2) = subplot(3,2,2);

imhist(Igray);

hist = imhist(Igray);

maxhist = max(hist);

ylim([0 maxhist]); % Scale y axis scale

grid on;

title('2. Histogram of Gray Image intensity')

M = Igray > 2;

Igray(~M) = 0;

ax(4) = subplot(3,2,3);

imshow(M);

title('3. Intensity Mask')

ax(5) = subplot(3,2,4);

imshow(Igray);

title('4. Mask + Image')

ax(3) = subplot(3,2,5);

% Create 2D countour map

%sperate figure that changes colorbar scale based on user defined

%injection levels

cont = contourf(Igray);

l = length(levelList);

[C,h] = contourf(Igray, levelList);

c = colorbar;

% colormap(hot);

title('5. Injection Contours')

set(gca, 'YDir','reverse')

daspect([1 1 1])

colorbar('Ticks',levelList,...

'TickLabels',injectionList)

ax(3) = subplot(3,2,6);

% Create 2D countour map

%sperate figure that changes colorbar scale based on user defined

%injection levels

Iblur2 = imgaussfilt(Igray,20);

cont2 = contourf(Iblur2, 'edgecolor', 'none');

c = colorbar;

% colormap(hot);

title('6. Blended Injection Contours')

set(gca, 'YDir','reverse')

daspect([1 1 1])

colorbar('Ticks',levelList,...

'TickLabels',injectionList)

% colormap(jet), shading interp

**Calculation of acoustic intensity and heterogeneity**

Using ImageJ software, the regions of interest (ROIs) corresponding to the deposited POL were selected using the freehand selection tool in the main menu bar. The analyze tool was used to obtain the image histogram of the selected ROIs. From the generated histogram analysis, the mean pixel intensity and standard deviation values were used to calculate acoustic intensity and acoustic heterogeneity, respectively.

**Optimization of %MBs in POL as feasibility study**

|  |
| --- |
| **Fig.S1. In vivo US and CT imaging of POL.** (A) Doppler ultrasound imaging to select injections sites with minimal vessels and depiction of needle (white arrows) pre-injection of POL. (B) Ultrasound imaging of hypoechoic deposition using 0.01% MBs in 4mL POL (red arrow). (C) Ultrasound imaging of hyperechoic deposition using 0.1%MBs in 4mL POL (red arrow). (E) 3D distribution of 4mL POL *in vivo* immediately post-injection (red), and 3h post procedure (green). (D) CT image post-injection of 4mL POL loaded with 40 mg/mL iodine where some leakage to and adjacent was present. (E) Post-mortem CT imaging and (F) post-mortem US of injected POL using 0.1% MBs. (G) The deposition gradually became hypoechoic in its center (white asterisk) with a hyperechoic margin (yellow arrow). |

**Multimodal Analysis of Post-Injection POL Distribution: Integrating CT, Ultrasound, and 3D Mapping Techniques**

**CT imaging**

| 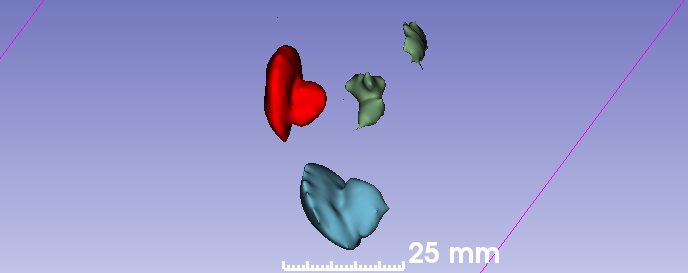 |
| --- |
| **Fig.S2.** **In vivo 3D distribution of POL in kidney.** Three injections of POL with SEHN in swine kidney depicted with red, blue, and green colors. Green colors depict an injection that produced leaked material. |

|  |
| --- |
| **Fig.S3. In vivo morphometrical measurements of POL after injection with three needle devices.** (A) Area, major axis, and minor axis of POL injected with SEHN (n=3) and calculated from CT imaging coronal plane. (B) Areas of POL depositions injected with MPIN (n=3), (C) MSHN (n=3), and (D) MPIN (IM) (n=3). Circularity and solidity of POL depositions injected with (E) SEHN (n=3), (F) MPIN (n=3), (G) MSHN (n=3), and (H) MPIN (IM) and calculated from CT imaging coronal plane. |

**Short-term *In vivo* imaging and pharmacokinetic study of poloxamer-based formulation with DOX (POLDOX), and formulation without DOX (DOXSoln)**

|  |
| --- |
| **Fig.S4.** **In vivo iodine distribution of POL.** 1D relative iodine distribution over normalized distance for POL injected with (A) SEHN (n=4), (B) MSHN (n=3), (C) MPIN (n=3), and (D) MPIN (IM) (n=3). (E) AUC of relative concentration of iodine over distance for each needle device. *p<0.05 from one-way ANOVA statistical test. |

|  |
| --- |
| **Fig.S5. In vivo US imaging of POL post-mortem.**US imaging of POL injections with (A) SEHN and (B) MPIN (IM) after tissue explanation. With arrows point hypoechoic POL depositions. |

**Serial growth of POL injection with SEHN**

|  |
| --- |
| **Fig.S6. 3D representation of POL injected into the liver, leaking towards the hepatic artery, and zoom of POL (upper image) going towards the hepatic vein (down).** |

**Short-term *In vivo* imaging and pharmacokinetic study of poloxamer-based formulation with DOX (POLDOX), and formulation without DOX (DOXSoln)**

|  |
| --- |
| **Fig.S7. In vivo study of POLDOX 3D distribution in the liver per mL injected.** (A) 3D Sphericity and solidity of 4 mL POLDOX injected with SEHN. (B) 3D growing pattern of POLDOX injected at 10 mL/h with leaked material. (C) 3D growing pattern of localized POLDOX injected at 10 mL/h. Error bars depict standard deviations from mean values (n=2 for 1, 2 and, 3 mL; n=3 for 4 mL), Statistical tests were performed with one-way ANOVA. |

|  |
| --- |
| **Fig.S8**. **In vivo 1D distribution of iodine in POLDOX over time.** 1D relative concentration of iodine over normalized distance for POLDOX at (A) 0 min, (B) 30 min, (C) 60 min, (D) 120 min, (E) 180 min, and (F) 240 min. Each color depicts different swine. |

|  |
| --- |
| **Fig.S9. AUC of relative iodine concentration of iodine over normalized distance for POLDOX for different timepoints**. AUCs were calculated from Fig.S8. Error bars depict standard deviations from mean values (n=3 for 0 min, and n=2 for 30 min to 240 min). One-way ANOVA statistical tests were performed. |

|  |
| --- |
| **Fig.S10. Major and minor axes of POLDOX post-injection over time.** Error bars depict standard deviations from mean values (n=3, 0 min to 180 min; n=2, 240 min). |

|  |
| --- |
| **Fig.S11. Areas of the ranges of iodine concentration at for POLDOX over time.** (A) 0, (B) 30, (C) 60, (D) 120, (E)180, and (F) 240 min. Error bars depict standard deviations from mean values (n=3 for 0 min, and n=2 for 30 min to 240 min). *p<0.05, **p<0.01, ***p<0.001 from one-way ANOVA statistical test. |

| **Table S1. 3D morphometrics and radiopacity of POL injections in swine as a function of needle devices.** | | | | | | |
| --- | --- | --- | --- | --- | --- | --- |
| **Device** | **Volume (mL)** | **Surface area (cm^2^)** | **Sphericity (a.u)** | **Solidity (a.u)** | **Hounsfield units (a.u)** | **SA/V (cm^-1^)** |
| SEHN (n=4) | 4.2 ±0.3 | 14.5 ±1.9 | 0.9 ±0.1 | 0.8 ±0.2 | 468.8 ±64.0 | 3.5 ±0.3 |
| MPIN (n=3) | 3.8 ±0.7 | 26.8 ±3.9 | 0.44 ±0.0 | 0.2 ±0.0 | 279.7 ±3.4 | 7.0 ±0.6 |
| MSHN (n=3) | 1.1 ±0.2 | 10.6 ±2.5 | 0.53 ±0.1 | 0.4 ±0.4 | 349.6 ±116.2 | 8.9 ±2.4 |
| MPIN (IM) (n=3) | 5.2 ±0.2 | 29.8 ±4.1 | 0.49 ±0.0 | 0.5 ± 0.1 | 426.0 ±30.5 | 5.7 ±0.6 |
| ± depicts standard deviations from mean value. SA/V, surface area to volume ratio. | | | | | | |

| **Table S2. Areas of relative iodine concentration distributions in tissue after 4mL POL injection** | | | | | | |
| --- | --- | --- | --- | --- | --- | --- |
|  | **Relative iodine concentration** | | | | | |
| **Needle device** | **7 – 13 mg/mL** | | **13 – 27 mg/mL** | | **27 – 40 mg/mL** | |
|  | **Area** | **CV** | **Area** | **CV** | **Area** | **CV** |
| SEHN (n=4) | 0.4 ±0.1 cm^2^ | 26.2 | 0.4 ±0.1 cm^2^ | 36.8 | 2.0 ±0.7 cm^2^ | 36.8 |
| MSHN (n=3) | 0.8 ±0.5 cm^2^ | 61.3 | 0.8 ±0.5 cm^2^ | 66.9 | 0.1 ±0.1 cm^2^ | 94.0 |
| MPIN (n=3) | 0.9 ±0.3 cm^2^ | 41.2 | 0.8 ±0.4 cm^2^ | 48.3 | 0.5 ±0.5 cm^2^ | 93.9 |
| MPIN (IM) (n=3) | 1.4 ±0.6 cm^2^ | 44.0 | 1.4 ±1.2 cm^2^ | 83.5 | 1.5 ±1.3 cm^2^ | 89.1 |
| + Depicts standard deviations. CV= Coefficient of variance. | | | | | | |

| **Table S3. Comparison of 2D morphometrical analysis of needle devices with US and CT imaging.** | | | | | |
| --- | --- | --- | --- | --- | --- |
| **Needle** | **Parameter** | **US imaging** | **CV** | **CT imaging** | **CV** |
| SEHN | Area | 2.3 ±0.3 | 12.6 | 2.6 ±0.5 | 18.6 |
|  | Major axis | 2.4 ±0.3 | 11.8 | 2.3 ±0.2 | 10.0 |
|  | Minor axis | 1.4 ±0.2 | 18.4 | 1.5 ±0.1 | 10.7 |
|  | Circularity | 0.3 ±0.1 | 24.1 | 0.9 ±0.0 | 1.7 |
|  | Solidity | 0.7 ±0.1 | 8.1 | 1.0 ±0.0 | 0.3 |
|  | Perimeter | 10.0 ±1.2 | 12.3 | 6.0 ±0.5 | 9.0 |
| MSHN | Area | 1.4 ±0.5 | 40.6 | 1.3 ±0.6 | 49.0 |
|  | Major axis | 2.9 ±0.5 | 17.7 | 2.5 ±0.3 | 13.3 |
|  | Minor axis | 1.4 ±0.2 | 33.0 | 1.5 ±0.4 | 16.1 |
|  | Perimeter | 8.8 ±1.9 | 21.2 | 6.7 ±1.3 | 19.1 |
| MPIN * | Area | - | - | 1.2 ±0.0 | 3.0 |
|  | Major axis | - | - | 3.0 ±0.3 | 10.6 |
|  | Minor axis | - | - | 1.8 ± 0.4 | 24.6 |
|  | Perimeter | - | - | 9.1 ±1.1 | 12.2 |
| MPIN (IM) | Area | 3.6 ±0.2 | 5.8 | 2.6 ±0.3 | 33.0 |
|  | Major axis | 2.7 ±0.7 | 26.6 | 3.5 ±1.3 | 38.2 |
|  | Minor axis | 1.6 ±0.4 | 23.2 | 2.0 ±0.3 | 24.6 |
|  | Perimeter | 9.1 ±1.1 | 12.4 | 12.0 ±1.2 | 10.4 |
| * US imaging measurements were not included for MPIN because they were not readily visible. MPIN measurements from CT imaging are from small depositions in from of beads that left a trace while prongs were retracting. ± Depicts standard deviations. CV= Coefficient of variance. n=3. | | | | | |

| **Table S4. 3D morphological measurements of POL injected with SEHN and imaged with US, and CT.** | | | | |
| --- | --- | --- | --- | --- |
| **Parameter** | **US imaging** | **CV** | **CT imaging** | **CV** |
| Volume (mL) | 11.3 ±4.8 | 42.4 | 4.2 ±0.3 | 8.4 |
| Sphericity (a.u) | 0.7 ±0.1 | 11.6 | 0.9 ±0.1 | 9.0 |
| Solidity (a/u) | 0.6 ±0.0 | 8.7 | 0.8 ±0.2 | 20.9 |
| SA/V (cm^-1^) | 7.3 ±1.3 | 18.4 | 3.5 ±0.3 | 8.1 |
| n=3 for US imaging, n=4 for CT imaging. ± Depicts standard deviations. CV= Coefficient of variance. | | | | |

| **Table S5. 3D and 2D morphometrics of POL** | | | | | | | | | | | | |
| --- | --- | --- | --- | --- | --- | --- | --- | --- | --- | --- | --- | --- |
|  | **3D** | | **2D** | | | | | | | | | |
|  | **Sphericity (a.u)** | **Solidity (a.u)** | **Area (cm^2^)** | | **Major axis (cm)** | | **Minor axis (cm)** | | **Circularity (a.u)** | | **Solidity (a.u)** | |
| **Injected volume (mL)** | **CT** | **CT** | **US** | **CT** | **US** | **CT** | **US** | **CT** | **US** | **CT** | **US** | **CT** |
| 1 | 0.8 ±0.1 | 0.6 ±0.3 | 1.1 ±0.4 | 0.6 ±0.2 | 1.6 ±0.4 | 1.0 ±0.2 | 1.1 ±0.4 | 0.6 ±0.1 | 0.5 ±0.1 | 0.9 ±0.1 | 0.8 ±0.1 | 1.0 ±0.0 |
| 2 | 0.8 ±0.1 | 0.8 ±0.2 | 1.5 ±0.6 | 1.3 ±0.3 | 1.7 ±0.5 | 1.6 ±0.2 | 1.4 ±0.5 | 0.9 ±0.1 | 0.3 ±0.1 | 0.9 ±0.0 | 0.8 ±0.0 | 1.0 ±0.0 |
| 3 | 0.8 ±0.1 | 0.8 ±0.2 | 1.8 ±0.9 | 1.7 ±0.7 | 2.1 ±0.8 | 1.8 ±0.4 | 1.4 ±0.6 | 0.9 ±0.2 | 0.3 ±0.2 | 0.9 ±0.0 | 0.7 ±0.1 | 1.0 ±0.0 |
| 4 | 0.8 ±0.1 | 0.8 ±0.2 | 1.9 ±1.2 | 2.0 ±1.0 | 2.1 ±0.9 | 1.9 ±0.6 | 1.4 ±0.4 | 1.0 ±0.2 | 0.3 ±0.2 | 0.9 ±0.0 | 0.8 ±0.1 | 1.0 ±0.0 |
| n=3 for US imaging, n=4 for CT imaging. ± Depicts standard deviations. CV= Coefficient of variance. | | | | | | | | | | | | |

| **Table S6. CBCT imaging analysis of radiopacity, % of volume post-4mL injection and morphometrics of POLDOX** | | | | |
| --- | --- | --- | --- | --- |
| **Time post 4mL injection (min)** | **Hounsfield units (a.u)** | **Volume % post-4mL injection** | **Sphericity (a.u)** | **Solidity (a.u)** |
| 0 | 460.2 ±123.7 | 100 ±0.0 | 0.8 ±0.0 | 0.8 ±0.0 |
| 30 | 393.8 ±15.3 | 92.7 ±6.1 | 0.9 ±0.0 | 0.9 ±0.0 |
| 60 | 388.8 ±6.9 | 92.1 ±8.3 | 0.9 ±0.0 | 0.9 ±0.1 |
| 90 | 380.4 ±0.8 | 90.8 ±10.4 | 0.9 ±0.0 | 0.9 ±0.1 |
| 120 | 376.2 ±14.8 | 90.1 ±13.7 | 0.9 ±0.0 | 0.9 ±0.1 |
| 180 | 377.9 ±8.5 | 86.6 ±12.2 | 0.9 ±0.0 | 0.9 ±0.1 |
| 240 | 331.6 ±62.8 | 73.0 ±5.9 | 0.9 ±0.0 | 0.9 ±0.0 |
| n=3 for 0 min values, n=2 for 30- to 240- min values. ± Depicts standard deviations. | | | | |

| **Table S7. Summary of DOX levels in plasma for POLDOX (n=2) and DOXSOln (n=3).** | | | | | | |  |  |
| --- | --- | --- | --- | --- | --- | --- | --- | --- |
| Time | Cohort | | | | | |  |  |
| min | POLDOX (ng/mL) | | DOXSoln (ng/mL) | | | |  |  |
| 0.5 | 0.0 ± 0.0 | | 10.4 ±17.9 | | | |  |  |
| 2 | 31.9 ± 45.2 | | 540.7 ±420.9 | | | |  |  |
| 5 | 22.5 ± 31.8 | | 1667.2 ±986.8 | | | |  |  |
| 10 | 138.0 ± 195.3 | | 3405.9 ±1088.9 | | | |  |  |
| 20 | 1405.6 ± 1135.2 | | 5284.0 ±1681.3 | | | |  |  |
| 24 | 2052.2 ± 704.4 | | 4947.1 ±1015.1 | | | |  |  |
| 26 | 1936.7 ± 957.7 | | 2673.4 ±1450.7 | | | |  |  |
| 29 | 1480.1 ± 540.1 | | 2207.3 ±172.9 | | | |  |  |
| 34 | 603.5 ± 257.1 | | 1224.1 ±316.3 | | | |  |  |
| 44 | 185.0 ± 105.3 | | 543.3 ±198.1 | | | |  |  |
| 64 | 146.3 ± 71.4 | | 290.4 ±159.5 | | | |  |  |
| 84 | 132.1 ± 84.3 | | 136.9 ±118.6 | | | |  |  |
| 114 | 62.3 ± 4.3 | | 107.0 ±61.0 | | | |  |  |
| 144 | 104.6 ± 84.8 | | 48.9 ±45.4 | | | |  |  |
| 204 | 31.2 ± 5.0 | | 54.4 ±48.8 | | | |  |  |
| 264 | 39.7 ± 11.2 | | 32.8 ±56.9 | | | |  |  |
| **Table S8.** **Summary of DOX levels in organs and injection site for POLDOX (n=2) and DOXSOln (n=3). p values obtained from t-test statistical study.** | | | | | | | | |
| Formulation | | DOX levels in tissue (ng/mg of tissue) | | | | | | |
|  | | Heart | | Kidney | Liver | Spleen | | Injection site (liver) |
| POLDOX | | 0.0 ±2.0 | | 21.0 ±13.6 | 6.8 ±4.8 | 9.8 ±2.0 | | 680,000.0 ±339411.2 |
| DOXSoln | | 16.2 ±11.3 | | 55.4 ±7.7 | 12.6 ±1.2 | 23.3 ±2.8 | | 340000.0 ±240,208.2 |
| p values | | 0.1788 | | 0.0737 | 0.0412 | 0.0080 | | 0.2717 |
