# Supplementary material for: In vivo imaging and pharmacokinetics of percutaneously injected ultrasound and X-ray imageable thermosensitive hydrogel loaded with doxorubicin versus free drug in swine
Source: PLoS One. 2024 Dec 19;19(12):e0310345. doi: 10.1371/journal.pone.0310345 (PMC11658602; doi:10.1371/journal.pone.0310345)
Supplement: Attachment — Submitted filename: Response editor PLOSONE.docx [file pone.0310345.s003.docx]

Dear Dr. Isha Mutreja,

Academic Editor

PLOS ONE

We greatly appreciate your time in considering our manuscript with Submission ID PONE-D-24-37693 titled “In vivo Imaging and Pharmacokinetics of Percutaneously Injected Ultrasound and X-ray Imageable Thermosensitive Hydrogel loaded with Doxorubicin versus Free Drug in Swine”. Your guidance and the reviewer’s suggestions were highly appreciated to improve this manuscript.

Please find below point by point (in blue color) the responses for each point you kindly provided.

Thank you very much for considering our manuscript.

Sincerely,

Bradford J. Wood, MD.

Center for Interventional Oncology

Clinical Center

National Institutes of Health

Jose F. Delgado, PhD. Cand.

Center for Interventional Oncology

Clinical Center

National Institutes of Health

Fischell Department of Bioengineering

University of Maryland, College Park

1. Please ensure that your manuscript meets PLOS ONE's style requirements, including those for file naming. The PLOS ONE style templates can be found at

<https://journals.plos.org/plosone/s/file?id=wjVg/PLOSOne_formatting_sample_main_body.pdf> and

<https://journals.plos.org/plosone/s/file?id=ba62/PLOSOne_formatting_sample_title_authors_affiliations.pdf>

**Answer: The manuscript was adapted to the suggested PLOSOne format.**

2. Please note that PLOS ONE has spec6ific guidelines on code sharing for submissions in which author-generated code underpins the findings in the manuscript. In these cases, all author-generated code must be made available without restrictions upon publication of the work. Please review our guidelines at <https://journals.plos.org/plosone/s/materials-and-software-sharing#loc-sharing-code> and ensure that your code is shared in a way that follows best practice and facilitates reproducibility and reuse.

**Answer: Thank you for bringing this point, the codes were included in S1 Apprendix.**

3. We note that the grant information you provided in the ‘Funding Information’ and ‘Financial Disclosure’ sections do not match.

When you resubmit, please ensure that you provide the correct grant numbers for the awards you received for your study in the ‘Funding Information’ section.

**Answer: We incorporated the grant numbers in the Financial disclosure section of the manuscript.**

4. Thank you for stating the following financial disclosure:

"“This work was supported by the NIH Center for Interventional Oncology and the Intramural Research Program of the National Institutes of Health via intramural NIH Grants Z1A CL040015, 1ZIDBC011242."

Please state what role the funders took in the study.  If the funders had no role, please state: ""The funders had no role in study design, data collection and analysis, decision to publish, or preparation of the manuscript.""

If this statement is not correct you must amend it as needed.

Please include this amended Role of Funder statement in your cover letter; we will change the online submission form on your behalf.

**Answer: The role of funders was added as follows in the funding disclosure section: "The funders had no role in study design, data collection and analysis, decision to publish, or preparation of the manuscript."**

5. Thank you for stating the following in the Acknowledgments Section of your manuscript:

"This work was supported by the Center for Interventional Oncology in the Intramural Research Program of the National Institutes of Health (NIH) by intramural NIH Grants NIH Z01 1ZID BC011242 and CL040015 and by the National Cancer Institute grant 1ZIABC011313-09. J.F.D. was supported by the Jayne Koskinas  Ted Giovanis Foundation for Health and Policy through the NIH Graduate Partnership Program from the  Office of Intramural Training and Education (GPP-OITE). J.F.D. is currently a participant of the Graduate Partnership Program through the University of Maryland Fischell Department of Bioengineering in the A. James Clark School of Engineering."

We note that you have provided funding information that is not currently declared in your Funding Statement. However, funding information should not appear in the Acknowledgments section or other areas of your manuscript. We will only publish funding information present in the Funding Statement section of the online submission form.

Please remove any funding-related text from the manuscript and let us know how you would like to update your Funding Statement. Currently, your Funding Statement reads as follows:

"“This work was supported by the NIH Center for Interventional Oncology and the Intramural Research Program of the National Institutes of Health via intramural NIH Grants Z1A CL040015, 1ZIDBC011242."

Please include your amended statements within your cover letter; we will change the online submission form on your behalf.

**Answer: We removed the funding information from acknowledgements and add it to financial disclosure section.**

6. Thank you for stating the following in the Competing Interests section:

"BW is Principal Investigator on the following CRADA’s = Cooperative Research & Development Agreements, between NIH and industry: Philips (CRADA), Philips Research (CRADA), Celsion Corp (CRADA), BTG Biocompatibles / Boston Scientific) (CRADA), Siemens (CRADA), NVIDIA (CRADA), XAct Robotics (CRADA). ProMaxo (CRADA), Negotiating CRADA with Tempus, Galvanize, Theromics, Imactis, Canon Medical, Varian, MediView. NV is a staff Scientist from Philips Healthcare."

Please confirm that this does not alter your adherence to all PLOS ONE policies on sharing data and materials, by including the following statement: ""This does not alter our adherence to  PLOS ONE policies on sharing data and materials.” (as detailed online in our guide for authors <http://journals.plos.org/plosone/s/competing-interests>).  If there are restrictions on sharing of data and/or materials, please state these. Please note that we cannot proceed with consideration of your article until this information has been declared.

Please include your updated Competing Interests statement in your cover letter; we will change the online submission form on your behalf.

**Answer: “We added the following information to the conflicts of interest section: "This does not alter our adherence to PLOS ONE policies on sharing data and materials.”**

**We also removed: Uro 1 (CRADA), and Mediview (CRADA). Negotiating CRADA with Tempus, Galvanize, Theromics, Imactis, Canon Medical, Varian, MediView.**

7. PLOS requires an ORCID iD for the corresponding author in Editorial Manager on papers submitted after December 6th, 2016. Please ensure that you have an ORCID iD and that it is validated in Editorial Manager. To do this, go to ‘Update my Information’ (in the upper left-hand corner of the main menu), and click on the Fetch/Validate link next to the ORCID field. This will take you to the ORCID site and allow you to create a new iD or authenticate a pre-existing iD in Editorial Manager.

**Answer: Thank you, the ORCID iD for the corresponding author was updated.**

8. We note that you have included the phrase “data not shown” in your manuscript. Unfortunately, this does not meet our data sharing requirements. PLOS does not permit references to inaccessible data. We require that authors provide all relevant data within the paper, Supporting Information files, or in an acceptable, public repository. Please add a citation to support this phrase or upload the data that corresponds with these findings to a stable repository (such as Figshare or Dryad) and provide and URLs, DOIs, or accession numbers that may be used to access these data. Or, if the data are not a core part of the research being presented in your study, we ask that you remove the phrase that refers to these data.

**Answer: Thank you for bringing this important note, the following information was modified in the manuscript:**

**Line 514. “No drop in oxygen levels was observed during the injection procedure.”**

9. Please include captions for your Supporting Information files at the end of your manuscript, and update any in-text citations to match accordingly. Please see our Supporting Information guidelines for more information: <http://journals.plos.org/plosone/s/supporting-information>.

**Answer: The captions were added for supporting information files.**

10. Please review your reference list to ensure that it is complete and correct. If you have cited papers that have been retracted, please include the rationale for doing so in the manuscript text, or remove these references and replace them with relevant current references. Any changes to the reference list should be mentioned in the rebuttal letter that accompanies your revised manuscript. If you need to cite a retracted article, indicate the article’s retracted status in the References list and also include a citation and full reference for the retraction notice.

**Answer: The reference list was reviewed and they are correct.**
